# Supplementary material for: MSSA: multi-stage semantic-aware neural network for binary code similarity detection
Source: PeerJ Comput Sci. 2025 Jan 17;11:e2504. doi: 10.7717/peerj-cs.2504 (PMC11784775; doi:10.7717/peerj-cs.2504)

Raw Data

The code for MSSA is available at GitHub (https://github.com/SQAbin/MSSA)


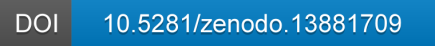


The screenshot of the experimental results is as follows:

1. BCSD results on classification performance


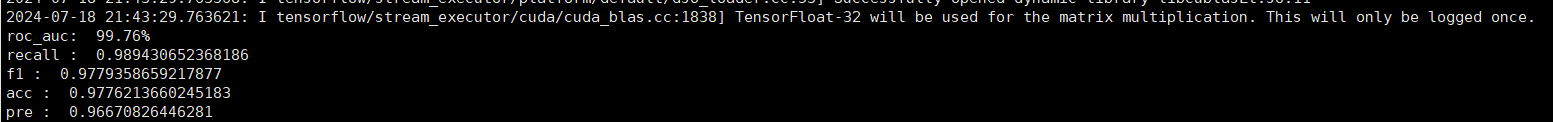


2. BCSD results on retrieval metric MRR and Recall@1


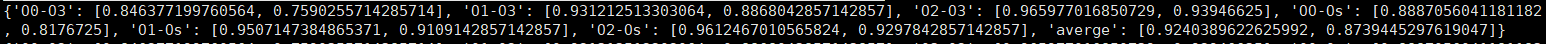


3. Results on complexity and efficiency

Number of Model Parameters


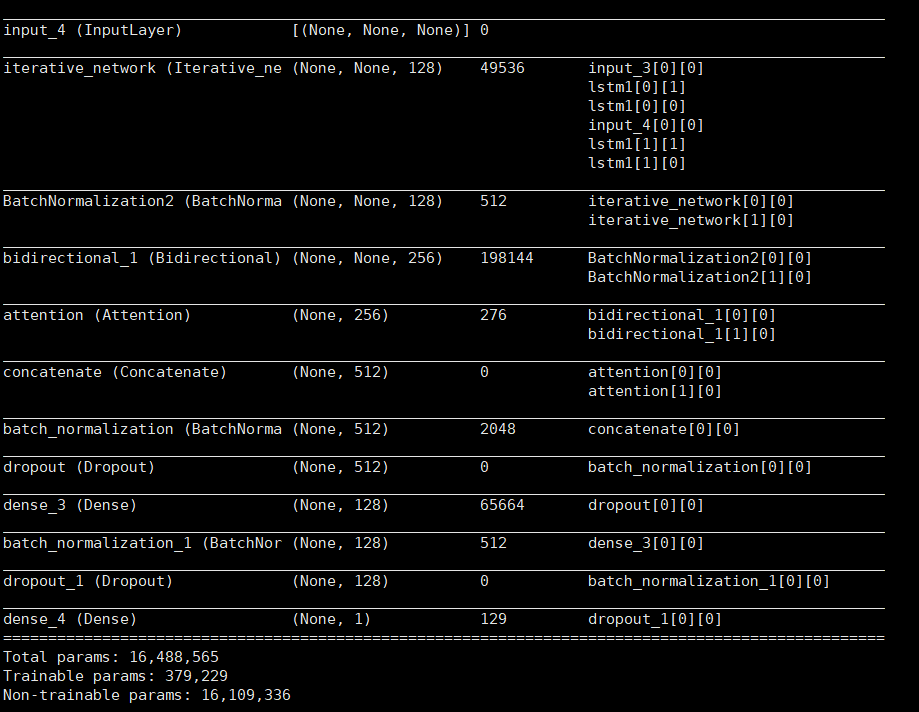


Execution Speed


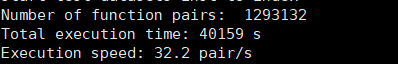


4. BCSD results on ablation study of MSSA

MSSA-NIB


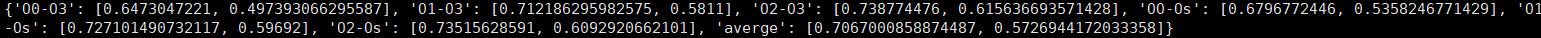


MSSA-NAM


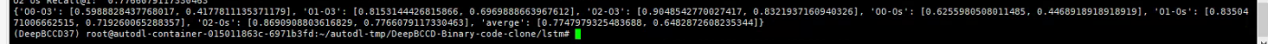


MSSA-NA


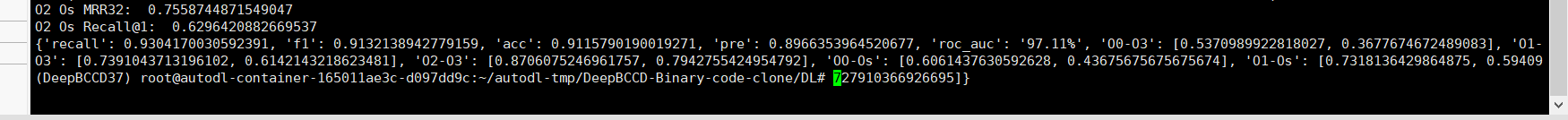

Supplement: Supplemental Information 1 [file peerj-cs-11-2504-s001.docx]
